# Forward and reverse genetic dissection of morphogenesis identifies filament-competent *Candida auris* strains

**DOI:** 10.1038/s41467-021-27545-5

**Published:** 2021-12-10

**Authors:** Darian J. Santana, Teresa R. O’Meara

**Affiliations:** Department of Microbiology and Immunology, University of Michigan Medical School, Ann Arbor, MI USA

**Keywords:** Fungal biology, Fungal genetics, Cellular microbiology, Pathogens

## Abstract

*Candida auris* is an emerging healthcare-associated pathogen of global concern. Recent reports have identified *C. auris* isolates that grow in cellular aggregates or filaments, often without a clear genetic explanation. To investigate the regulation of *C. auris* morphogenesis, we applied an *Agrobacterium*-mediated transformation system to all four *C. auris* clades. We identified aggregating mutants associated with disruption of chitin regulation, while disruption of *ELM1* produced a polarized, filamentous growth morphology. We developed a transiently expressed Cas9 and sgRNA system for *C. auris* that significantly increased targeted transformation efficiency across the four *C. auris* clades. Using this system, we confirmed the roles of *C. auris* morphogenesis regulators. Morphogenic mutants showed dysregulated chitinase expression, attenuated virulence, and altered antifungal susceptibility. Our findings provide insights into the genetic regulation of aggregating and filamentous morphogenesis in *C. auris*. Furthermore, the genetic tools described here will allow for efficient manipulation of the *C. auris* genome.

## Abstract (web summary)

Some isolates of the emerging fungal pathogen *Candida auris* can form cellular aggregates or filaments. Here, Santana and O’Meara use *Agrobacterium*-mediated transformation and a CRISPR-Cas9 system to identify several genes that regulate *C. auris* morphogenesis.

## Introduction

Since its 2009 isolation from the ear canal of a patient in Japan, the emerging fungal pathogen *Candida auris* has caused infections and outbreaks in at least 44 countries on 6 continents^1^. The global prevalence of *C. auris* is characterized by the seemingly simultaneous emergence of four distinct genetic clades, differing on the scale of hundreds of thousands of single nucleotide polymorphisms (SNPs), with a potential fifth clade recently identified^2,3^. Individual isolates exhibit significant heterogeneity both within and between clades, including in murine models of infection and colonization^4,5^. The continually increasing understanding of biologically and clinically relevant phenotypic variation among *C. auris* isolates, and the variation between *C. auris* and other well-studied model organisms, emphasizes the need for facile genetic manipulation approaches to allow for mechanistic characterization of this organism.

Although *C. auris* does not form filaments under many of the same environmental cues that induce hyphal growth in *Candida albicans*^6^, numerous reports of irregular or multicellular growth indicate *C. auris* does exhibit cellular polymorphism. Depletion of the essential molecular chaperone *HSP90* results in elongated cell growth^6^. Genotoxic stress induced by hydroxyurea or deletion of the DNA damage responsive long non-coding RNA DINOR similarly result in pseudohyphal elongated cells^7,8^. Other stressors such as growth in high salt concentrations induce cell elongation^9^. Strains exhibiting filamentous, elongated, or aggregating morphologies have been isolated from populations of *C. auris* cells following murine infection^10,11^. Furthermore, numerous reports detail patient isolates with multicellular aggregating phenotypes, often described by a failure of cell aggregates to disperse upon mixing or vortexing^12–15^. Aggregating isolates exhibit reduced biomass in biofilm formation and lower virulence in *Galleria mellonella* infection models compared to non-aggregating counterparts^12,16^. Still, the genetic determinants of irregular morphogenesis in *C. auris* remain largely unexplored due in part to difficulties in performing genetic manipulation in this organism.

Transformation of *C. auris* is complicated by low rates of targeted integration and variable transformation efficiency among isolates and clades^7,17^. The use of RNA−protein complexes of purified Cas9 and gene-specific guide RNAs, referred to as Cas9-ribonucleoproteins (RNPs), to promote homology directed repair demonstrably increases transformation efficiency and targeted integration rates^18^. Transformation incorporating RNPs is often the method of choice for manipulating the *C. auris* genome, and variations exist using multiple gRNA target sites to further improve targeted integration efficiency^19^. The use of RNPs in transformation, however, comes with increased expense and additional technical considerations during transformation. In *C. albicans*, transformation with linearized gene cassettes encoding Cas9 and sgRNA promote homozygous gene deletion; these cassettes cannot be detected in the genome of transformants, suggesting they are transiently expressed and not stably integrated^20^. A similar transiently expressed CRISPR-Cas9 system promotes targeted genetic manipulation in *Cryptococcus neoformans*^21^. We hypothesized that specific adaptation of the transiently expressed CRISPR-Cas9 system to use *C. auris*-recognized promoters would increase the rates of targeted transformation efficiency.

A forward genetics system represents an alternative approach for manipulating the genome. The piggyBac transposon mutagenesis system has proven successful for performing insertional mutagenesis at saturating levels in a Clade II *C. auris* isolate^8^. This represents a significant advance in the technical ability to genetically manipulate *C. auris*. However, one potential limitation of the piggyBac system is that it requires initial engineering of the strain of interest to encode the transposon machinery prior to performing genome-scale mutagenesis. To develop a forward genetics system suitable for performing mutagenesis in any *C. auris* clinical isolate without prior engineering, we turned to *Agrobacterium tumefaciens-*mediated transformation (AtMT), an insertional mutagenesis approach with a history of proven success in fungal species^22^. *A. tumefaciens* is a plant pathogen that causes crown gall in dicotyledonous plants through genetic transformation^23^. Its capacity for transformation is not limited to plants, however, and can be taken advantage of to perform insertional transformation in a variety of eukaryotic species, including *C. albicans*, *Candida glabrata*, and *Saccharomyces cerevisiae*^24^. In practice, mobilization of a DNA sequence flanked by left and right direct repeats (T-DNA) is accomplished by induction of *A. tumefaciens* virulence genes during co-culture with a recipient organism using acetosyringone^25^. This T-DNA sequence is encoded on the Ti Plasmid harbored by *A. tumefaciens* and can be manipulated to contain fungal selectable markers.

We used AtMT to generate an insertional mutant library in *C. auris* and identified morphogenic mutants exhibiting aggregating or filamentous growth. Insertions in genes orthologous to regulators of chitinase and chitin synthase in *S. cerevisiae* were associated with defects in daughter cell separation in *C. auris*, leading to aggregating growth, while an insertion in an ortholog of *ScELM1* resulted in constitutive filamentous growth in *C. auris*. We developed a robust transient CRISPR-Cas9 expression system for *C. auris* and demonstrated its ability to significantly increase targeted transformation in isolates from all four major clades. Using this system, we performed deletions in key regulators of cell separation to demonstrate functional conservation of *ELM1* and chitin regulatory genes as morphogenic regulators in *C. auris*. The morphogenic mutants we identified exhibited attenuated virulence in a *G. mellonella* infection model and altered antifungal susceptibility profiles. The tools presented here allowed for detailed analyses of the genetic circuitry required for morphogenesis in the emerging pathogen *C. auris* and will serve as a resource to the community for future molecular genetic manipulation of this pathogen.

## Results

### *Agrobacterium-*mediated transformation identifies *C. auris* morphogenic mutants

**Fig. 1:**
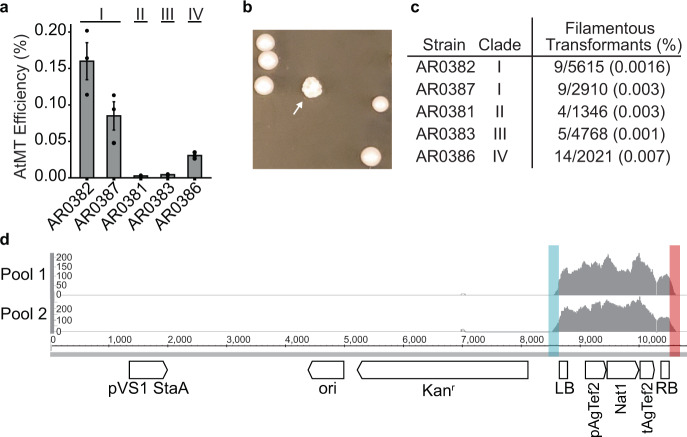
*Agrobacterium tumefaciens*-mediated transformation (AtMT) identifies regulators of colony morphology in *C. auris*. **a** AtMT transformation efficiency of *C. auris* was measured for representative isolates from each of four major clades. Transformation efficiency is expressed as the ratio of recovered *C. auris* transformants to the total number of input *C. auris* cells. Data shown are mean ± standard error of the mean from three independent experiments. Source data are provided as a Source Data file. **b** Morphogenic mutants were identified in *C. auris* AtMT transformants through irregular colony morphologies (arrow). **c** The rates of morphogenic mutants recovered for insertional mutants from representative isolates from each of four major clades are expressed as the ratio of morphogenic mutant CFU to total CFU screened for aberrant morphology. **d** Genomic DNA was extracted from 6 morphogenic mutants from AR0382 (Clade I) and pooled into two pools of 3 for Illumina sequencing. Reads were mapped to the TI Plasmid (pTO128), represented as a read coverage plot. Highlighted regions in blue and red indicate read sequence that extended beyond the T-DNA left and right borders, respectively, used to identify transgene insertion sites in the *C. auris* genome.

While aggregating and filamentous strains of *C. auris* have been recovered from human and murine hosts, the genetic circuitry governing *C. auris* morphogenesis remains largely uncharacterized. Therefore, we set out to apply a forward genetic approach to identify regulators of morphogenesis in *C. auris*. To accomplish this, we developed an AtMT system for *C. auris*. We cloned the CaNAT1 nourseothricin resistance cassette into the pPZP Ti plasmid backbone between the T-DNA left and right borders to generate pTO128 (pPZP-NATca) and transformed the resulting vector into *A. tumefaciens* strain EHA105, which also harbors the virulence genes necessary for mobilization of the T-DNA. We used representative *C. auris* clinical isolates from the FDA-CDC Antimicrobial Resistance Isolate Bank^26^ to measure the transformation efficiency of AtMT in each of the four major *C. auris* clades (Fig. 1a). By comparing the number of recovered nourseothricin-resistant transformants to the number of input cells, we observed successful transformation in isolates from all four clades with variable transformation efficiency. Of the isolates tested, we observed the highest transformation efficiency in the Clade I isolate AR0382, with an average efficiency of 0.16% (1 in 625 *C. auris* cells) (Fig. 1a). The Clade II isolate AR0381 showed the lowest transformation efficiency at 0.0025% (1 in 40,000 *C. auris* cells) under the same growth conditions, though even this rate is consistent with the range of transformation efficiencies exhibited in integrative AtMT of other yeast species (Fig. 1a)^27,28^. We visually screened recovered transformants for those with altered colony morphology, suggestive of an alteration in cellular morphology (Fig. 1b). In this manner, we identified morphological mutants in isolates from all four major clades. The rate at which we recovered morphological mutants differed significantly among the clades (Chi sq. = 22.42, *p* = 1.66 × 10^−4^), with the highest rate in the Clade IV isolate AR0386 (Fig. 1c). These findings demonstrate the utility of AtMT as a forward genetics system to discover mutant phenotypes in all four major clades of *C. auris*.

Transgene insertion sites can be defined by identifying the genomic regions flanking the insertions using whole-genome sequencing^29^. We reasoned a similar approach could identify transgene insertion sites from multiple mutants sequenced in pools. We mapped Illumina sequencing reads from two pools of three morphogenic mutants selected from AtMT of AR0382 (Clade I) to the sequence of the TI plasmid pTO128 (pPZP-NATca). The sequencing reads mapped exclusively to the T-DNA region of the plasmid, demonstrating the specificity of the integration, with additional read length spanning either junction at the T-DNA left and right borders (Fig. 1d). The sequence extending beyond the left and right borders corresponded to *C. auris* genomic regions flanking the transgene insertions. We deconvoluted the pools using standard PCR and Sanger sequencing with insertion site-specific primers.

**Fig. 2:**
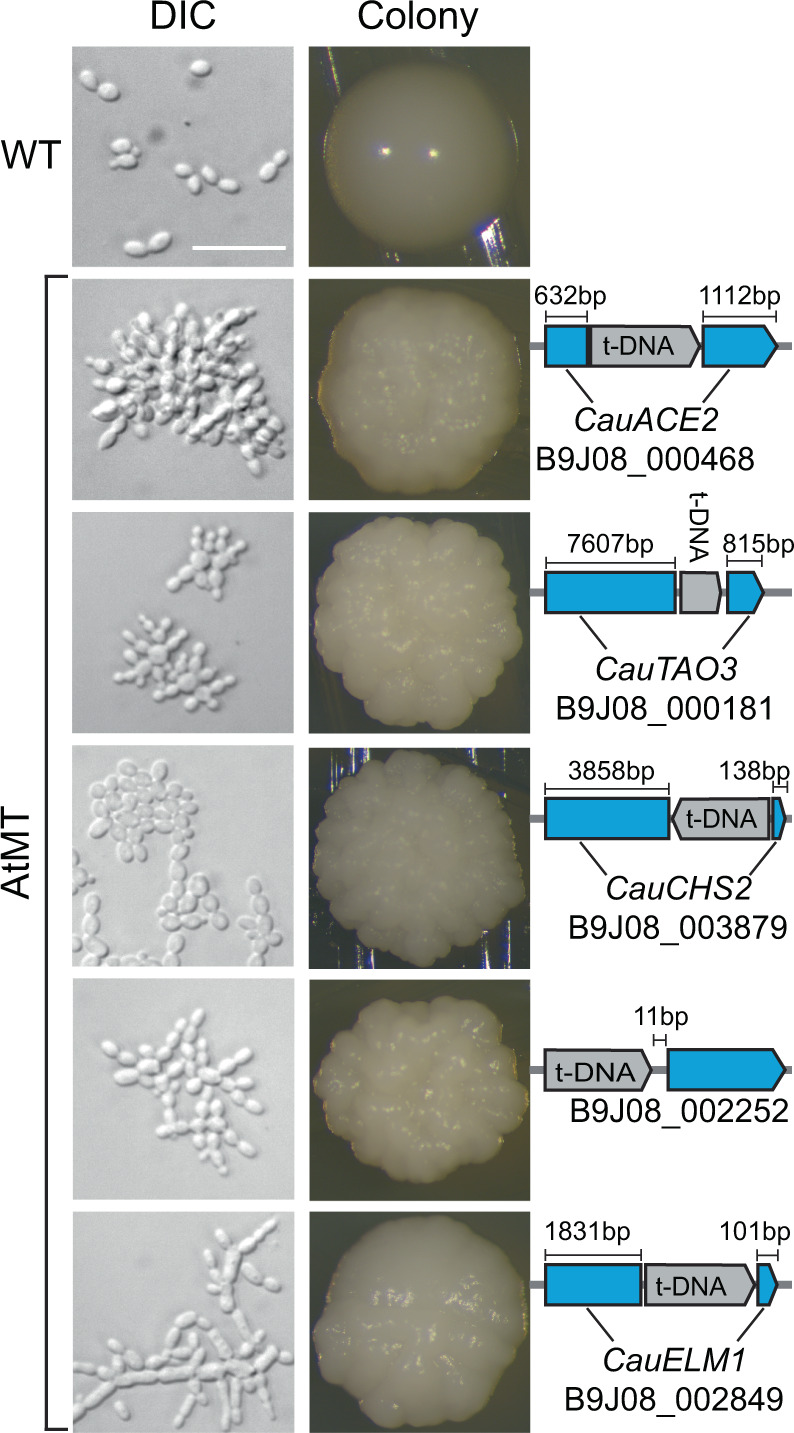
Transgene insertion sites associated with *C. auris* morphogenic mutants. Cell (DIC microscopy, Differential Interference Contrast) and colony morphologies demonstrate distinct morphogenic defects in five AtMT (*Agrobacterium tumefaciens*-mediated transformation) insertional mutants (bottom) compared to wild-type *C. auris* AR0382 (top). Identified transgene insertion sites were confirmed using Sanger sequencing (right). In all five cases, T-DNA insertion events were not accompanied by any additional insertions or deletions in the insertion locus. Scale bar = 20 μm.

Among the mutants identified by irregular colony morphologies, four exhibited a similar aggregating cellular phenotype, with individual cells connected into clusters that could not be disrupted by vortexing (Fig. 2). Insertion events in *CauACE2* (B9J08_000468), orthologous to *S. cerevisiae ACE2* (YLR131C), as well as in *CauTAO3* (B9J08_000181), orthologous to *S. cerevisiae TAO3* (YIL129C), were associated with this aggregatory phenotype. A similar aggregating phenotype resulted from an insertion near the C-terminus of *CauCHS2* (B9J08_003879), an ortholog of *CHS2* (YBR038W) in *S. cerevisiae*. A fourth aggregating strain was associated with an insertion in the upstream region of *B9J08_002252*; however, orthologs of this gene in related species are poorly characterized. To predict a potential function for this gene, we analyzed the *C. albicans* ortholog *C7_00260C* using the CalCEN Co-expression network^30^. GO term analysis revealed that 43 of 50 co-expressed genes fall under the “piecemeal microautophagy of the nucleus” term (Supplementary Fig. 1). We also observed pseudohyphae-like filaments characterized by elongated cells with constricted separations between compartments in a mutant with an insertion in *CauELM1* (B9J08_002849), an ortholog of *S. cerevisiae ELM1* (YKL048C) (Fig. 2).

A sixth insertional mutant identified by its irregular colony morphology exhibited similar aggregating growth (Supplementary Fig. 2). For this mutant, we identified T-DNA sequence both in the intergenic space upstream of the B9J08_002954 ORF and in the intergenic region upstream of the B9J08_002667 ORF from the B8441 reference sequence, but we were unable to amplify the complete insertion locus of either site from genomic DNA of the mutant. We hypothesize that a recombination event or other chromosomal rearrangement may have occurred following one or multiple T-DNA insertion events in this mutant, though further investigation is required to confirm this. Together, these findings identify key components of the regulation of cell separation in *C. auris*.

### Expression of Cas9 and sgRNA increases targeted integration in *C. auris*

**Fig. 3:**
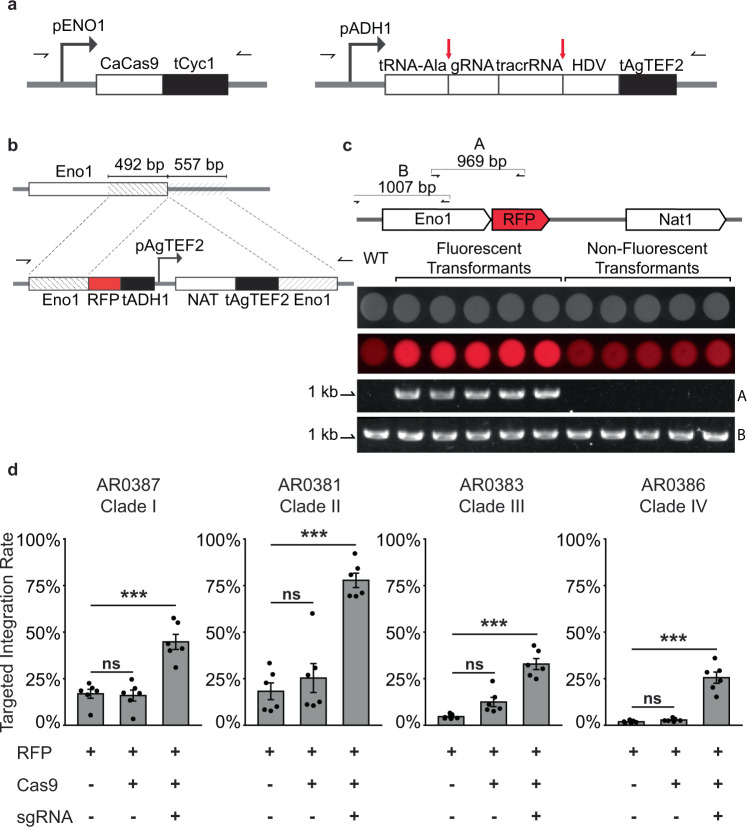
A CRISPR-Cas9 expression system promotes targeted transformation in four *C. auris* clades. **a** Structures of the Cas9 and sgRNA expression cassettes. *CAS9* is driven by the *C. auris ENO1* promoter and followed by the *CYC1* terminator. The sgRNA cassette is driven by the *C. auris ADH1* promoter and contains *C. auris* tRNA-Ala immediately upstream of the 20-bp gRNA sequence and hepatitis delta virus (HDV) ribozyme immediately downstream of the tracrRNA sequence. Predicted post-transcriptional cleavage sites are indicated by red vertical arrows. Primer sites to generate linear transformation cassettes are indicated by horizontal arrows. **b** Design of the reporter cassette for measuring targeted integration. The cassette is flanked by approximately 500-bp homology to the *C. auris ENO1* C-terminus minus the stop codon and the region immediately downstream of *C. auris ENO1*. *RFP* and the *C. auris ADH1* terminator tag the *ENO1* gene at the C-terminus via a glycine linker to generate *ENO1-RFP* in targeted transformants. An independently driven nourseothricin resistance cassette (NAT) allows identification of total transformants, regardless of integration site, by selection with nourseothricin. **c** Targeted integration events are identifiable by colony fluorescence. Transformation of AR0387 was performed using the reporter cassette described in panel (**b**). Representative fluorescent transformants and non-fluorescent transformants were spotted onto YPD. Primer set A, spanning the *ENO1-RFP* junction, shows amplification only from fluorescent transformants. Primer set B, spanning a neighboring wild-type locus, shows amplification from all transformants and the wild type. **d** Expression of Cas9 and sgRNA promotes targeted integration rate. Transformation was performed in representative isolates from all four major *C. auris* clades with the linear transformation cassettes described in panels (**a**) and (**b**). Transformations were performed with and without Cas9 and sgRNA elements; when absent, the cassettes were replaced with an equivalent volume of buffer. Targeted integration rate is expressed as the ratio of fluorescent colonies recovered to total nourseothricin-resistant colonies recovered. Each point represents an individual transformation. Shown are the mean ± standard error of the mean from three individual experiments, each performed in duplicate. Source data are provided as a Source Data file. Statistical differences were determined using one-way ANOVA with Tukey’s post hoc test for multiple comparisons: AR0387: ***p = 5.4 × 10^−5^, ns: *p* = 0.98; AR0381: ***p = 5.8 × 10^−6^, ns: *p* = 0.66; AR0383: ***p = 6.0 × 10^−7^, ns: *p* = 0.063; AR0386: ***p = 3.0 × 10^−7^, ns: *p* = 0.92.

To validate the insertional mutagenesis and confirm the role of identified genes in regulating the multicellular phenotypes we observed, we sought to recapitulate the phenotypes via clean deletions of the target genes. However, targeted homologous recombination has low efficiency in *C. auris*, adding considerable technical challenge to performing genetic manipulation^7,17^. Transformation in *C. auris* can be facilitated by the use of Cas9 and sgRNA ribonucleoproteins; however, a previous DNA-based transient CRISPR-Cas9 expression approach used in *C. albicans* does not substantially improve targeted transformation efficacy in *C. auris* (ref. ^6^ and personal communication, Sang Hu Kim). Recently, Ng and Dean^31^ reported variable increases in targeted transformation efficiency in *C. albicans* when using different promoters to drive the transcription of the sgRNA in a similar system. We hypothesized that the low efficiency of the transient CRISPR system in *C. auris* may be due to poor recognition of the *SNR52* promoter from *C. albicans*. Therefore, we sought to develop a transient Cas9 and sgRNA expression system that can be used for efficient transformation in *C. auris*^20,31^. First, we generated expression cassettes for Cas9 and sgRNA using *C. auris*-specific promoters (Fig. 3a). We placed the CaCas9 cassette, which has been codon-optimized for expression in CTG clade fungi, under control of the *C. auris ENO1* promoter and the sgRNA cassette under control of the *C. auris ADH1* promoter. However, because this RNA Polymerase II promoter would generate a transcript with a 5′ cap and 3′ polyA tail, ultimately detrimental to the gRNA targeting efficiency, we included the *C. auris tRNA-ALA* sequence immediately upstream of the sgRNA and the hepatitis delta virus (HDV) ribozyme sequence immediately downstream of the sgRNA. With this design, we anticipated cleavage at the 3′ end of the tRNA sequence by endogenous RNase A and self-catalyzed cleavage at the 5′ end of the HDV ribozyme^32,33^.

To assess the functional capacity of the Cas9 and sgRNA expression system to increase the efficiency of targeted integration in *C. auris*, we designed a reporter cassette that would allow for rapid and specific identification of targeted integration events (Fig. 3b). The reporter cassette contained approximately 500 bp of homology to the C-terminus of *C. auris ENO1* and genomic sequence immediately downstream of *ENO1*. We removed the stop codon from the *ENO1* C-terminus homologous sequence and fused *RFP* to the *ENO1* C-terminus with a glycine linker. Because the *RFP* gene had no promoter element, we anticipated transformants would only demonstrate robust fluorescence if the reporter cassette integrated precisely in frame to tag the Eno1 protein and be driven by the endogenous *ENO1* promoter. The reporter cassette also included an independently driven nourseothricin resistance (NAT) cassette to allow identification of the total transformant population by selection on nourseothricin, regardless of integration site. To confirm that the reporter cassette specifically identified targeted integration events, we designed a PCR primer set spanning the *ENO1-RFP* junction and a primer set spanning a region of the *ENO1* locus native to the wild type. We performed transformation with the reporter cassette and recovered a representative sample of nourseothricin-resistant transformants that were either fluorescent or non-fluorescent. Amplification of the region spanning the *ENO1-RFP* junction was only exhibited by the fluorescent transformants and not by the wild-type or non-fluorescent transformants, while amplification of the wild-type sequence was exhibited by all the transformants and the wild-type strain (Fig. 3c). This demonstrates that the ratio of fluorescent to non-fluorescent colonies is a reliable measure of the efficiency of targeted integration.

We observed variable targeted transformation efficiency among *C. auris* isolates of different genetic backgrounds (Fig. 3d). We therefore sought to determine whether the Cas9 and sgRNA expression system promoted targeted transformation in multiple genetically diverse *C. auris* isolates (Fig. 3d). The targeted integration rate under each transformation condition was determined by dividing the number of fluorescent colonies by the total number of nourseothricin-resistant transformant colonies. For AR0387, a Clade I isolate, inclusion of the Cas9 and sgRNA expression cassettes increased the targeted integration rate to 44.7% of transformants from an average of 16.9% using only the reporter cassette. We observed similar trends for isolates from each clade. Targeted integration increased from 18.2 to 77.8% in AR0381 (Clade II), from 4.6 to 32.8% in AR0383 (Clade III), and from 1.8 to 25.5% in AR0386 (Clade IV) with the addition of the Cas9 and sgRNA expression cassettes compared to the reporter cassette alone (Fig. 3d). The *ENO1* C-terminus homologous arm encoded by the reporter cassette showed 100% sequence identity in all four isolates, while AR0381 and AR0383 shared four nucleotide variants out of 557 bp in the downstream homologous arm and AR0386 showed a single nucleotide variant in the same region (Supplementary Fig. 3). Therefore, differences in the targeted integration efficiency could not be explained by differential homology to the reporter cassette. Moreover, we were unable to detect integration of the *CAS9* cassette in a majority of recovered fluorescent transformants across all four clades (Supplementary Fig. 4), suggesting the Cas9 system is transient as designed, with rare integration events, consistent with previous observations from a similar system in *C. albicans*^20^. Taken together, these observations indicate the Cas9 and sgRNA expression cassettes successfully promote targeted transformation in all four major *C. auris* clades.

### *ACE2* and *TAO3* are regulators of *C. auris* morphogenesis

**Fig. 4:**
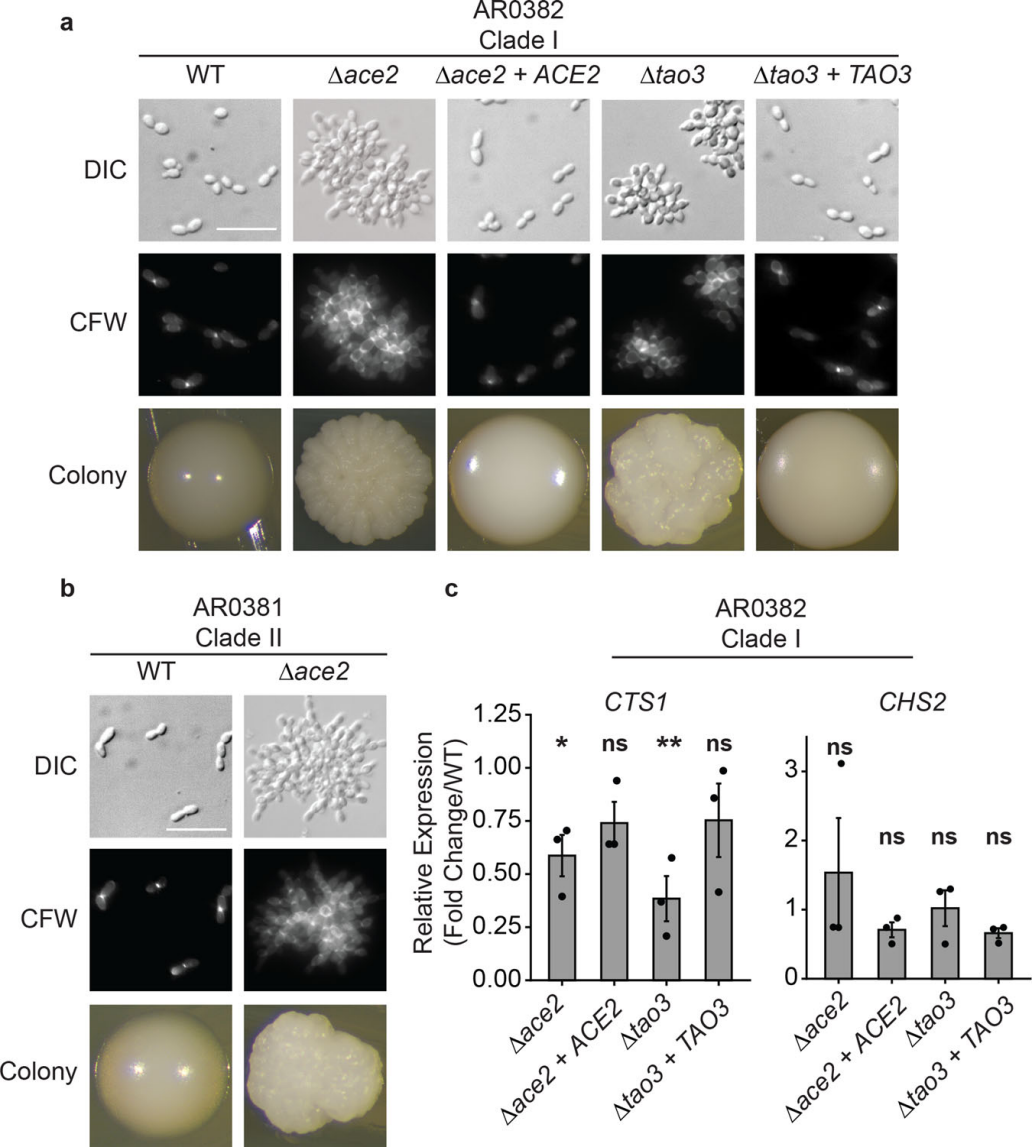
*ACE2* and *TAO3* are regulators of *C. auris* morphogenesis. **a** Microscopy of *Δace2*, *Δtao3*, and complemented strains in the AR382 (Clade I) genetic background. Representative images shown for DIC (Differential Interference Contrast), cells stained with calcofluor white (CFW), and colonies formed on YPD agar. Scale bar = 20 μm. **b** *ACE2* regulates morphogenesis across *C. auris* clades. Microscopy of *Δace2* and in the AR381 (Clade II) genetic background. Representative images shown for DIC, cells stained with calcofluor white, and colonies formed on YPD agar. Scale bar = 20 μm. **c** *ACE2* and *TAO3* regulate putative chitinase *CTS1* but not *CHS2* transcription. Wild-type (AR0382), *Δace2*, *Δtao3*, and complemented strains were grown to exponential phase in YPD at 30 °C prior to RNA extraction and RT-qPCR analysis of upregulated and downregulated genes. Shown are the relative expression of *CTS1* and *CHS2* for each mutant strain compared to the wild type and normalized to *ACT1* gene expression. Mean ± standard error of the mean from three biological replicates, each with three technical replicates. Strains that showed significantly different expression compared to the wild type are indicated. Source data are provided as a Source Data file. Statistical differences were determined using one-way ANOVA with Dunnett’s post hoc test for multiple comparisons. *CTS1*: *Δace2*, *p *+= 0.046; *Δace2* + *ACE2*, ns: *p* = 0.25; *Δtao3*, **p = 0.004; *Δtao3* + *TAO3*, ns: *p* = 0.32. *CHS2*: *Δace2*, ns: *p* = 0.87; *Δace2* + *ACE2*, ns: *p* = 0.60; *Δtao3*, ns: *p* = 0.93; *Δtao3* + *TAO3*, ns: *p* = 0.10.

Using these tools, we were able to investigate the function of the genes implicated in *C. auris* morphogenic regulation by AtMT. Deletion of *ACE2* or *TAO3* in AR0382 (Clade I) resulted in constitutively aggregating cells with individual cells connected at septa, suggestive of a failure of budding daughter cells to separate from mother cells (Fig. 4a). We then codon-optimized a G418 resistance gene for CTG-clade expression and found its expression allowed for selection of *C. auris* on media containing 1 mg/mL G418. Using this new dominant selectable marker, we were able to complement *Δace2* and *Δtao3* mutants with reconstituted versions of the deleted genes replaced in the endogenous loci. The complemented strains *Δace2* + *ACE2* and *Δtao3* + *TAO3* restored the wild-type cellular and colony morphologies (Fig. 4a). In *S. cerevisiae*, Tao3 associates with kinases Kic1 and Cbk1 as part of the Regulation of *ACE2* Morphogenesis (RAM) pathway. Phosphorylation of Ace2 by Cbk1 results in its accumulation in daughter cell nuclei, where it regulates the expression of enzymes that mediate septum degradation^34^. Mutations in *ACE2* or upstream components of the RAM pathway in *S. cerevisiae* or in *C. albicans* result in a aggregating, multicellular phenotypes similar to those exhibited by *C. auris Δace2* and *Δtao3* mutants, suggesting that *C. auris* has maintained conservation of the RAM pathway in regulating morphogenesis^35–37^. An *Δace2* mutant in AR0381 (Clade II) showed a similar aggregating phenotype, suggesting this role is conserved across *C. auris* clades as well (Fig. 4b). To assess whether the regulation of cell wall maintenance genes was also conserved in *C. auris*, we investigated the transcriptional change in the chitinase gene *CTS1* (B9J08_002761), which is homologous to a key enzyme regulated by Ace2 and responsible for the degradation of the primary septum during daughter cell separation in *S. cerevisiae*^38^. We observed significant downregulation of *CTS1* expression in *Δace2* and *Δtao3* mutants compared to wild-type AR0382, while *Δace2* + *ACE2* and *Δtao3* + *TAO3* mutants showed no significant change in *CTS1* expression (Fig. 4c). Because our forward genetics screen suggested disruption of the chitin synthase gene *CHS2* could also confer an aggregating phenotype, we asked whether *CHS2* expression was altered by deletion of *ACE2* or *TAO3*. However, we observed no significant difference in the expression of *CHS2* in *Δace2* or *Δtao3* mutants compared to the wild type (Fig. 4c). Together, these findings demonstrate that *ACE2* and *TAO3* are key regulators of *C. auris* morphogenesis and deletion of either leads to an aggregating phenotype associated with decreased expression of the chitinase gene *CTS1*.

### *ELM1* is a regulator of *C. auris* filamentous growth

**Fig. 5:**
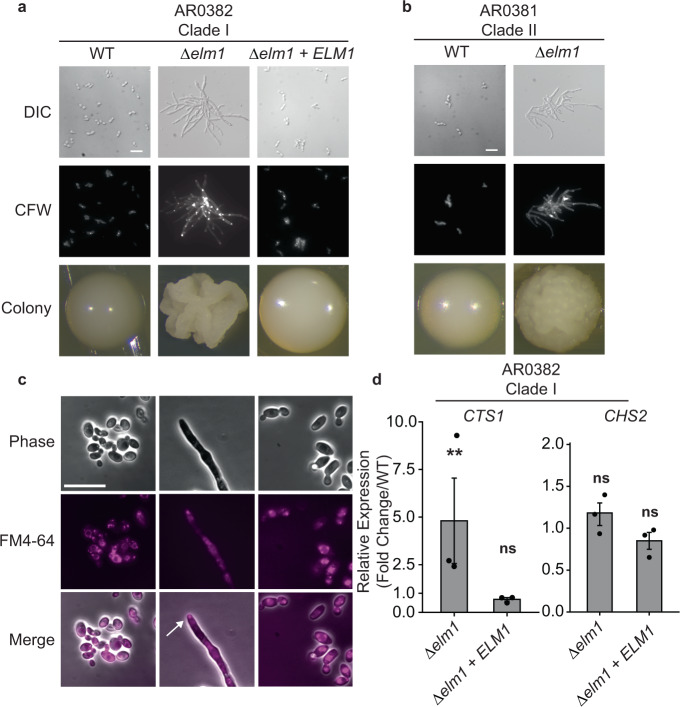
*ELM1* is a regulator of *C. auris* filamentous growth. **a** Microscopy of wild-type AR0382, *Δelm1*, and complemented *Δelm1* + *ELM1* strains. DIC (Differential Interference Contrast), cells stained with calcofluor white (CFW), and colonies formed on YPD agar are shown. Scale bar = 10 μm. **b** *ELM1* regulates filamentous growth across *C. auris* clades. Microscopy of DIC, cells stained with calcofluor white, and colonies formed on YPD agar for wild-type AR0381 (Clade II) and *Δelm1* in the AR0381 background. Scale bar = 10 μm. **c** *C. auris Δelm1* filaments form a Spitzenkörper-like structure at the filament apex. AR0382 (Clade I) wild type, *Δelm1*, and *Δelm1* + *ELM1* strains shown with phase contrast microscopy, stained with the lipophilic dye FM 4−64. Arrow indicates the location of a putative Spitzenkörper where dye has accumulated at the growing filament tip. Scale bar = 10 μm. **d** *ELM1* negatively regulates *CTS1* but not *CHS2* expression. Wild-type (AR0382), *Δelm1*, and *Δelm1* + *ELM1* were grown to exponential phase in YPD at 30 °C prior to RNA extraction and RT-qPCR analysis of upregulated and downregulated genes. Shown are the relative expression of *CTS1* and *CHS2* for each mutant strain compared to the wild type and normalized to *ACT1* gene expression. Mean ± standard error of the mean from three biological replicates, each with three technical replicates. Strains that showed significantly different expression compared to the wild type are indicated. Source data are provided as a Source Data file. Statistical differences were determined using one-way ANOVA with Dunnett’s post hoc test for multiple comparisons. *CTS1*: *Δelm1*, **p = 0.0014; *Δelm1* + *ELM1*, ns: *p* = 0.35. *CHS2*: *Δelm1*, ns: *p* = 0.98; *Δelm1* + *ELM1*, ns: *p* = 0.24.

Next, we investigated *ELM1*, disruption of which resulted in both an aggregating and elongated cellular phenotype in our AtMT screen. Deletion of *ELM1* in AR0382 (Clade I) resulted in a polarized growth phenotype resembling filaments (Fig. 5a). Individual cells remained conjoined at invaginated junctions, forming elongated compartments with similar widths to the wild-type yeast cells. Complementation with the wild-type *ELM1* gene restored the wild-type budding yeast morphology (Fig. 4a). Deletion of *ELM1* in AR0381 (Clade II) resulted in a similar phenotype as deletion in AR0381 (Clade I), suggesting that its role in regulating polarized growth is conserved across clades (Fig. 5b).

Because filamentous growth has not been shown to be a natural phenotype in *C. auris*, we asked whether the filamentous growth exhibited by *Δelm1 C. auris* was driven by mechanisms consistent with natural filamentous growth in other fungal species. Maintenance of filamentous growth in other fungal species is achieved through the formation of a Spitzenkörper, a complex of vesicles that coordinates cell wall synthetic enzymes and actin cytoskeleton and related proteins at the apical tip of the growing filament^39^. We observed a structure at the apex of the growing *Δelm1* cells that took up the lipophilic dye FM 4−64, consistent with the formation of a Spitzenkörper (Fig. 5c). This structure appeared to be unique to the filamentous form of AR0382, as no similar polar structure was observed in the budding yeast wild-type or *Δelm1* + *ELM1* strains (Fig. 5c).

We next investigated whether the conjoined cell phenotype in AR0382 *Δelm1* was associated with alterations in *CTS1* or *CHS2* regulation like the aggregating mutants. We found the chitinase gene *CTS1* to be significantly upregulated in *Δelm1* compared to wild-type AR0382, while the chitin synthase gene *CHS2* was not significantly differentially expressed (Fig. 5d). The complemented strain *Δelm1* + *ELM1* showed no significant variation in the expression of *CTS1* or *CHS2* compared to wild-type AR0382 (Fig. 5d). Together, these findings implicate *ELM1* as a regulator of *C. auris* pseudohyphal growth.

### *C. auris* morphogenic mutants exhibit attenuated virulence and altered antifungal susceptibility

**Fig. 6:**
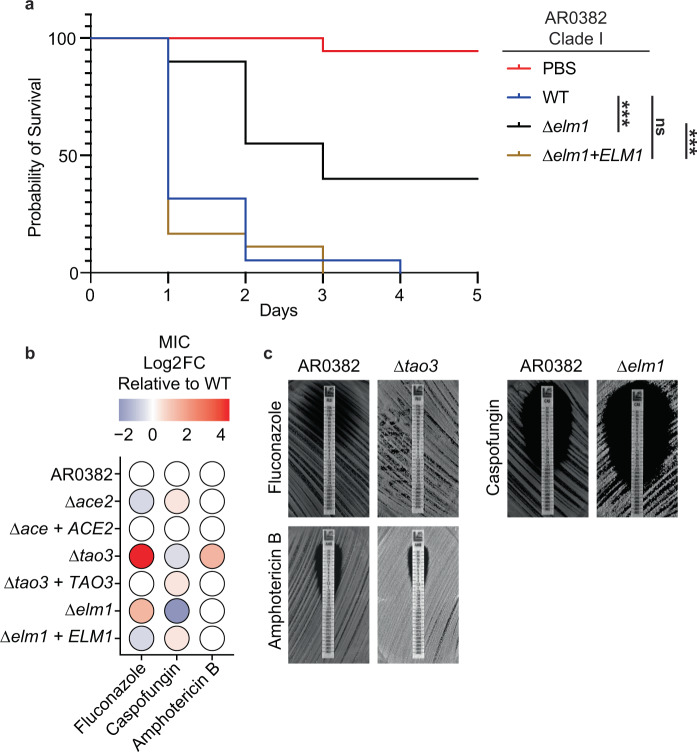
*C. auris* morphogenic mutants show attenuated virulence and altered antifungal susceptibility. **a** Wild-type AR0382 (Clade I), *Δelm1*, and *Δelm1* + *ELM1* strains in the AR0382 background were standardized to an optical density of OD_600_ = 1.0 in PBS before inoculating 20 *Galleria mellonella* larvae per *C. auris* strain with 50 μL of prepared inoculum. Larvae were maintained at 37 °C and monitored daily for survival for 5 days. Source data are provided as a Source Data file. Statistical differences were determined using a Mantel−Cox log-rank test. WT-*Δelm1*, ***p < 0.0001; WT-*Δelm1* + *ELM1*, ns: *p* = 0.51; *Δelm1-Δelm1* + *ELM1*, ***p < 0.0001. **b** Gradient MIC test strips were used to determine the susceptibility of wild-type AR0382, morphogenic mutants, and complemented strains in the AR0382 background to fluconazole, caspofungin, and amphotericin B. The color and intensity of each point corresponds to the Log_2_ fold change in MIC for each strain relative to the wild type. A complete list of MICs is available in Supplementary Table 1. **c** MIC test strips with zones of inhibition for mutants with substantially altered antifungal susceptibilities. *Δtao3* exhibited reduced susceptibility to fluconazole and amphotericin B compared to the wild type. *Δelm1* exhibited increased susceptibility to caspofungin.

Reports of *C. auris* isolates that exhibit aggregating or elongated cell morphologies have largely suggested these morphogenic variants show reduced virulence in infection models compared to budding yeast wild-type isolates^8,11,12^. We therefore investigated the pathogenic potential of the *Δace2, Δtao3*, and *Δelm1* mutants in a *Galleria mellonella* model of infection. The *Δelm1* strain exhibited the strongest attenuation of virulence compared to the parental AR0382 strain, with mortality rates recapitulating wild-type levels in the complemented *Δelm1* + *ELM1* strain (Fig. 6a). The *Δtao3* mutant did not exhibit significantly attenuated virulence compared to the parental AR0382 (*p* = 0.0563, Log-rank Mantel−Cox test), but the *Δace2* mutant showed a modest decrease in virulence (Supplementary Fig. 5a, b). Complemented strains encoding *ACE2* or *TAO3* genes showed similar mortality profiles to wild type AR0382 (Supplementary Fig. 5a, b).

We also investigated whether mutations in *ACE2, TAO3, or ELM1* were associated with altered antifungal susceptibility profiles. While the *Δace2* mutant did not exhibit a large difference in susceptibility to fluconazole, caspofungin, or amphotericin B compared to wild-type AR0382, the *Δtao3* and *Δelm1* mutants showed differential susceptibility profiles (Fig. 6b). The *Δtao3* mutant exhibited reduced sensitivity to fluconazole (MIC > 256 mg/L compared to 12 mg/L for WT AR0382) and amphotericin B (MIC 1 mg/L compared to 0.38 mg/L for WT AR0382) (Fig. 6c). The *Δelm1* mutant exhibited increased sensitivity to caspofungin (MIC 0.023 mg/L compared to 0.094 mg/L for WT AR0382) (Fig. 6c). In general, the complemented strains closely mimicked the susceptibility profile of the wild type (Fig. 6b). A complete list of MICs for each strain against fluconazole, caspofungin, and amphotericin B is available in Supplementary Table 1. Together, these findings demonstrate mutations in *C. auris ACE2, TAO3*, and *ELM1* are associated with altered virulence and antifungal susceptibility profiles in addition to altered morphogenesis.

## Discussion

We have developed approaches to performing facile, cost-effective forward and reverse genetic manipulation in *C. auris*. Using these tools, we identified functional conservation of chitinase regulatory pathways, disruption of which results in aggregating, multicellular growth in *C. auris*. We also uncovered a *C. auris* filamentous mutant, *Δelm1*, demonstrating the ability of *C. auris* to sustain filamentous growth. Our work represents part of a growing global effort to understand the biology of this emerging pathogen by offering alternative methods of improving its genetic tractability. We demonstrated the utility of AtMT for performing insertional mutagenesis in all four major *C. auris* clades without any prior engineering of the *C. auris* strain. We also demonstrated the ability of a *C. auris* CRISPR-Cas9 expression system to consistently and significantly improve targeted integration of a transformation cassette in representative isolates from all four major *C. auris* clades. Targeted integration rates were increased to levels at which mutants of interest can readily be identified by PCR or phenotypic screening. While this level of efficiency was associated with approximately 500 bp arms of homology, we successfully performed deletion of *TAO3* using a transformation cassette with only 50–70 bp of homology, albeit with reduced targeted transformation efficiency. Furthermore, we developed a new, codon-optimized G418 selectable marker for use in *C. auris*. Our work, in concert with similar advancements such as successful resistance marker recycling and piggyBac genome-wide transposon mutagenesis in *C. auris*^8,19,40^, will promote improved accessibility to mechanistic understanding of the genetic machinery in *C. auris*.

From our work, we identified *CauACE2* to be a key regulator of morphogenesis. In *S. cerevisiae*, *ACE2* daughter cell nuclear localization is regulated by the RAM pathway Kic1-Cbk1 kinase complex^34^. *ScTAO3*, sometimes called *PAG1*, physically associates with both *Sc*Kic1 and *Sc*Cbk1 and may mediate activation of Cbk1 by Kic1 ^41,42^. Disruption of *ScTAO3* or downstream *ScACE2* results in cellular aggregates and a failure of daughter cells to separate from mother cells during budding^38,41,42^. We observed similar aggregating phenotypes in *Δace2* and *Δtao3* mutants in *C. auris*. We therefore propose functional conservation of *ACE2* and the RAM regulatory pathway in *C. auris*. Downstream of this pathway, we identified a putative chitinase, *CauCTS1* (B9J08_002761), that was downregulated in *ΔCauace2* and *ΔCautao3* compared to the wild type. The sequence of *CauCTS1* contains no GPI-anchor signal sequence, and so is likely more closely related functionally to the secreted chitinases *ScCTS1* in *S. cerevisiae* and its functional homolog *CaCHT3* in *C. albicans* than to *CaCHT2* in *C. albicans*^43^. The regulation of *CauCTS1* by *CauACE2* is consistent with homologous pathways in *S. cerevisiae* and *C. albicans*, in which chitin degradation in the primary septum is mediated by the *ACE2*-regulated *Sc*Cts1 or *Ca*Cht3 proteins^36,44^. Interestingly, an experiment performing laboratory evolution of *S. cerevisiae* in a bioreactor resulted in multicellular, fast-sedimenting strains that were associated with mutations in *ACE2* ^35^. The design of the bioreactors in this example may have provided a selective advantage for multicellular growth due to increased sedimentation rate of cell aggregates compared to planktonic cells. An environmental niche may exist that produces a similar selective pressure against the regulatory network upstream of *CTS1* by offering a selective advantage for aggregating cells. Constitutively aggregating strains of *C. auris* have been isolated from clinical samples^12–15^. Based on publicly available RNA-seq data investigating this natural aggregating phenotype, we found that one such aggregating *C. auris* isolate exhibited significantly downregulated expression of *CTS1* compared to a non-aggregating counterpart when grown planktonically (log_2_FC = −1.3221, *p* = 6e−13)^15^. This observation is consistent with the mechanism of aggregation observed in the *Δace2* and *Δtao3* mutants, though we could not find evidence based on the RNA-seq data that the change in *CTS1* regulation of the natural aggregating isolate was directly related to the RAM pathway. Further characterization of the environmental reservoirs for *C. auris* may offer additional insight regarding the selective pressures driving similar phenotypes.

While the role of the serine-threonine kinase *ELM1* in regulating polar bud growth and morphogenic differentiation in *S. cerevisiae* has been long understood, its role in pathogenic fungi is largely unexplored^45,46^. One report demonstrated that deletion of *CgELM1* in *C. glabrata* results in moderately elongated cell growth, though this strain fails to recapitulate the fully pseudohyphal phenotype exhibited by *S. cerevisiae* or *C. auris*^47^. We observed elongated cells growing in pseudohyphal chains associated with an insertion event near the C-terminus of *CauELM1*. However, the full *Δelm1 C. auris* strains exhibited a more filamentous cell morphology. This discrepancy in cell morphologies between insertional disruption and clean deletion of *elm1* in *C. auris* may be explained by similar observations in *S. cerevisiae*. In *S. cerevisiae*, deletion of the C-terminal domain (aa 421−640 of 640) of *ELM1* results in increased Elm1 kinase activity, suggesting this domain may have autoinhibitory function^48^. This phenotype is associated with pseudohyphal growth with a cell morphology distinct from that demonstrated by *ΔScelm1*^48^. The distinct but similarly pseudohyphal phenotypes associated with the *C. auris ELM1* insertional mutant encoding a T-DNA insertion 101 base-pairs upstream of the *ELM1* C-terminus, but putatively encoding an intact kinase domain, and *ΔCauelm1* suggests similar *ELM1* regulation may exist in *C. auris*, though the extent to which the *ELM1* protein structure is altered for the insertion mutant is unclear. Intriguingly, the filamentous *Δelm1 C. auris* mutant exhibited a significant increase in the expression of the chitinase gene *CTS1* compared to the wild type. This is in contrast to *Δelm1* in *C. glabrata*, which exhibited decreased expression of *CgCTS1* compared to wild type^47^. Further characterization of Elm1 in diverse fungal species may yet reveal substantial variation in its function. The role that increased *CTS1* expression in *ΔCauelm1* plays in contributing to filamentous growth is unclear. One report indicated reduced expression of the *CTS1* homolog *CaCHT3* in hyphal *C. albicans* compared to *C. albicans* grown in the yeast form^49^. However, total chitinase activity was increased in *C. albicans* hyphae compared to yeast^50^. Whether *C. auris* filamentous growth is controlled by a similar chitinase function as *C. albicans* hyphal growth remains to be determined.

In phenotypic analysis, we observed alterations in virulence for the morphogenic mutants, consistent with other published reports^8,11,12^. We were also interested to observe altered antifungal susceptibility profiles associated with these mutants which may hint at larger roles for the genes of interest. In particular, the *Δtao3* mutant showed markedly reduced susceptibility to fluconazole and amphotericin B that appeared to be independent of *ACE2*, as the *Δace2* mutant did not show the same altered susceptibilities. The resistance to fluconazole conferred by deletion of *TAO3* may indicate the *C. auris* RAM pathway upstream of *ACE2* has divergent regulatory roles from the same pathway in *C. albicans*, mutation of which confers hypersensitivity to fluconazole^51^. Similarly, *C. albicans ace2Δ/ace2Δ* cells exhibit increased resistance to fluconazole that was not observed for *C. auris Δace2* cells^52^. While the full mechanistic contribution of the *C. auris* RAM pathway to antifungal susceptibility remains unclear, these observations highlight the possibility of regulatory network rewiring in *C. auris* and the importance of specifically investigating how *C. auris* has adapted regulatory pathways that may have well-established mechanisms in related model fungal species.

In sum, our work demonstrates an accessible approach to genetic engineering of *C. auris*, facilitating further understanding of the biology of this emerging pathogen. Using new forward and reverse genetic approaches, we characterized conserved and divergent key regulators of morphogenesis, virulence, and antifungal resistance in *C. auris*.

## Methods

### Strains and growth conditions

A list of *C. auris* and *A. tumefaciens* strains used in this study are listed in Supplementary Table 2. Unless specified otherwise, *C. auris* cells were grown at 30 °C in YPD liquid media (1% yeast extract, 2% peptone, 2% dextrose) with constant agitation. All strains were maintained in frozen stocks of 25% glycerol at −80 °C.

### Plasmids

A list of all plasmids used in this study is included in Supplementary Table 3. Constructs and sequences for pTO128, pTO135, pTO136, and pTO149 are available through Addgene (Watertown, MA, USA) under catalog #171105, #171103, #171104, and #177277.

A list of all primers used in this study is included in Supplementary Table 4.

#### pTO128

An *Agrobacterium* Ti-plasmid was constructed to include the CaNAT1 nourseothricin resistance cassette^53^ in the pPZP-NEO1 backbone^54^. The CaNAT1 cassette was excised at the *Sac*I and *Sal*I restriction sites from pLC49 ^55^ and ligated between the *Sac*I and *Sal*I restriction sites of pPZP-NEO1, replacing the G418 resistance cassette with CaNAT1 to form pTO128 (pPZP-NATca). pTO128 was subsequently electroporated into *A. tumefaciens* strain EHA 105^56^ using a Bio-Rad MicroPulser Electroporator.

All other transformation cassettes were maintained in the multiple cloning site of the pUC19 cloning vector^57^ and assembled from fragments as described below using the NEBuilder HIFI DNA Assembly master mix (NEB #E2621) according to the manufacturer’s instructions.

#### pTO135

To assemble pTO135 (pCauCas9), the Cas9 expression cassette minus the promoter sequence was PCR amplified from pLC963 ^58^ using primers oTO114-oTO115. A promoter region consisting of 1000 bp upstream of the *C. auris ENO1* gene (B9J08_000274) was PCR amplified from genomic DNA isolated from *C. auris* strain AR0387 using primers oTO112-oTO113. The pUC19 vector backbone was amplified using primers oTO116-oTO117.

#### pTO136

To assemble pTO136 (pCausgRNA), a promoter region consisting of 901 bp upstream of the *C. auris ADH1* gene (B9J08_004331) was PCR amplified using primers oTO118-oTO119 and assembled along with a synthesized DNA fragment (Genscript, Piscataway, NJ, USA) containing sequence from *C. auris* tRNA-Ala (B9J08_003096), a 20-bp gRNA sequence targeting the *ENO1* locus, a tracrRNA sequence, and an HDV ribozyme^31^. The pUC19 vector backbone was amplified using primers oTO120-oTO121.

#### pTO137

To assemble pTO137, containing the *ENO1-RFP* reporter cassette, the *RFP* construct was PCR amplified from pLC1047 ^59^ using primers oTO124-oTO125; a terminator sequence consisting of 933 bp downstream of the *C. auris ADH1* gene was PCR amplified from *C. auris* AR0387 genomic DNA using primers oTO126-oTO127; the CaNAT1 expression cassette including *TEF* promoter and terminator sequence was amplified from pLC49 using primers oTO128-oTO129; flanking regions containing homology to 492 bp at the C-terminal end of the *C. auris ENO1* gene minus the stop codon and 557 bp immediately 3′ of the *C. auris ENO1* gene were amplified from genomic DNA isolated from *C. auris* strain AR0387 genomic DNA using primers oTO122-oTO123 and oTO130-oTO131 respectively; the pUC19 vector backbone was amplified using primers oTO132-oTO133.

#### pTO154

To assemble pTO154 (p*ELM1::NAT*), 501 bp immediately 5′ of *ELM1* (B9J08_002849) and 502 bp immediately 3′ of *ELM1* were amplified from AR0387 genomic DNA using primers oTO317-oTO318 and oTO321-oTO337 respectively; the *CaNAT1* expression cassette was amplified from pLC49 using primers oTO319-oTO320; the pUC19 vector backbone was amplified using primers oTO323-oTO324.

#### pTO155

To assemble pTO155 (p*ACE2::NAT)*, 500 bp immediately 5′ of *ACE2* (B9J08_000468) and 498 bp immediately 3′ of *ACE2* were amplified from AR0387 genomic DNA using primers oTO325-oTO326 and oTO329-oTO330 respectively; the *CaNAT1* expression cassette was amplified from pLC49 using primers oTO327-oTO328; the pUC19 vector backbone was amplified using primers oTO331-oTO332.

#### pTO149

pTO149 was assembled to maintain the *NEO* (G418) resistance cassette. A codon-optimized *NEO* resistance gene was synthesized with every CUG codon replaced by the synonymous CUC leucine codon. This was assembled into pTO137 in place of the *NAT* resistance cassette, the backbone amplified with oTO272-oTO273. The codon-optimized *NEO* resistance gene was amplified including the *TEF* promoter and terminator sequence for assembly into complementation cassettes.

#### pTO169

To assemble pTO169 (p*ace2* + *ACE2*), the full-length *ACE2* gene along with 1001 bp upstream was amplified from AR0382 genomic DNA using oTO566-oTO567, the *ADH1* terminator sequence and the *NEO* resistance cassette were amplified from pTO149 with oTO568-oTO569, 832 bp downstream of *ACE2* was amplified from AR0382 genomic DNA using oTO570-oTO571, and the pUC19 backbone was amplified using oTO564-oTO565.

#### pTO174

To assemble pTO174 (p*tao3* + *TAO3*), the full-length *TAO3* gene along with 869 bp upstream was amplified from AR0382 genomic DNA using oTO584-oTO585, the *ADH1* terminator sequence and the *NEO* resistance cassette were amplified from pTO149 with oTO586-oTO587, 503 bp downstream of *TAO3* was amplified from AR0382 genomic DNA using oTO588-oTO589, and the pUC19 backbone was amplified using oTO582-oTO583.

#### pTO175

To assemble pTO175 (p*elm1* + *ELM1*), the full-length *ELM1* gene along with 997 bp upstream was amplified from AR0382 genomic DNA using oTO592-oTO593, the *ADH1* terminator sequence and the *NEO* resistance cassette were amplified from pTO149 with oTO594-oTO595, 582 bp downstream of *ELM1* was amplified from AR0382 genomic DNA using oTO596-oTO597, and the pUC19 backbone was amplified using oTO590-oTO591.

All *C. auris* genomic sequence data were obtained from the *C. auris* B8441 reference genome on *fungidb.org*^60^. All plasmid assemblies were verified by restriction digest and sanger sequencing.

### *Agrobacterium tumefaciens*-mediated transformation (AtMT)

AtMT was performed as previously described with minor modifications^61^. Briefly, *A. tumefaciens* strain EHA 105 harboring the pTO128 (pPZP-NATca) plasmid was cultured overnight at 30 °C in liquid Luria-Bertani (LB) media containing kanamycin. *A. tumefaciens* cells were harvested by centrifugation, washed once with sterile, ultrapure water, then resuspended at a final OD_600_ of 0.15 in liquid Induction Medium (IM) supplemented with 100 μM Acetosyringone 3′,5′-dimethoxy-4-hydroxyacetophenone (AS)^28^ and incubated at room temperature for 6 h with constant agitation. Recipient *C. auris* cells were harvested from an overnight culture grown at 30 °C in YPD by centrifugation then resuspended in sterile, ultrapure water at a final OD_600_ of 1.0. Equal volumes of prepared *A. tumefaciens* and *C. auris* were combined and the mixed culture was incubated on IM with AS agar at 23 °C for 4 days. Cells were then harvested into liquid YPD, washed three times with fresh YPD, then spread-plated onto YPD agar containing 200 μg/mL nourseothricin and 200 μg/mL cefotaxime. Plates were incubated at 30 °C for 2 days. Transformation efficiency was determined by dividing the total number of recovered *C. auris* CFU by the total input number of *C. auris* cells. To identify morphogenic mutants, colonies were screened visually for those exhibiting a wrinkled colony morphology then confirmed to exhibit aggregating or filamentous phenotypes using light microscopy.

### Genomic DNA isolation

Genomic DNA was isolated from *C. auris* morphological mutants to be used for downstream sequencing and insertion site mapping using a phenol-chloroform extraction. Briefly, cells were incubated overnight at 30 °C in liquid YPD then harvested by centrifugation and resuspended in breaking buffer (2% (v/v) Triton X-100, 1% (w/v) SDS, 100 mM NaCl, 10 mM Tris-Cl, 1 mM EDTA). DNA was extracted by bead beating into PCA then extracted into chloroform. Following precipitation by ethanol, extracted DNA was resuspended in TE buffer and treated with RNase A. Genomic DNA quality was assessed by 1% agarose gel electrophoresis.

### ATMT transgene mapping

Mapping of T-DNA insertion sites was performed similarly to methods previously described^29^. Genomic DNA isolated from six morphogenic mutants was collected and divided into two pools, each containing equal amounts by mass of genomic DNA from three individual mutants. Library preparation, quality control, and whole-genome sequencing were performed by Microbial Genome Sequencing Center (MIGS, Pittsburg, PA, USA). Library preparation was performed based on the Illumina Nextera kit and sequencing performed on the Nextseq 550 platform, generating 150 bp paired-end reads for each pool. Sequencing data were analyzed using the Galaxy web platform public server at *usegalaxy.org*^62^. Read quality was assessed using FASTQC and reads were trimmed using CutAdapt^63^ with a Phred quality cutoff of 20. A linearized vector reference sequence of pTO128 (pPZP-Natca) was generated from the circular vector sequence and 150 bp of sequence from the opposite border was added to each border of the linearized sequence. Reads were mapped to the linear pTO128 (pPZP-NATca) reference sequence using the Burrows–Wheeler Aligner with maximum exact matches (BWA-MEM)^64^ configured with default parameters except for minimum seed length = 50 and band width = 2. Mapped reads were visualized using IGV^62^ and sorted based on position and sequences that extended beyond the left and right boundaries of the tDNA was extracted. Consensus sequences of the extracted reads were mapped to the *C. auris* B8441 reference genome (GCA_002759435.2) using NCBI Blast. Primers specific to each identified insertion site were designed: oTO310 and oTO340 for *B9J08_002252*, oTO311 and oTO344 for *B9J08_003879*, oTO312 and oTO342 for *B9J08_002849*, oTO313 and oTO338 for *B9J08_000181*, oTO314 and oTO339 for *B9J08_000468*, oTO315 and oTO341 for *B9J08_002667*, and oTO316 and oTO343 for *B9J08_002954*. These were used to amplify the identified insertion regions in conjunction with T-DNA specific primers oTO6 and oTO90 using the genomic DNA from each of the six mutants as templates. Individual insertions were attributed to individual mutants based on amplicon length. Amplicons containing T-DNA insertions were Sanger sequenced to generate insertion maps for each mutant.

### *C. auris* transformation

Transformation of *C. auris* was performed as described previously, with minor modifications^18^. To generate *ENO1-RFP* strains, linear transformation cassettes encoding Cas9, sgRNA, and the RFP repair cassette were PCR amplified from pTO135, pTO136, and pTO137, respectively, using primers oTO18-oTO19. To generate *Δace2, Δelm1*, *Δace2* + *ACE2, Δtao3* + *TAO3*, and *Δelm1* + *ELM1* strains, a linear Cas9 cassette was amplified from pTO135 using primers oTO18-oTO19, linear repair cassettes were amplified from pTO155 for *ACE2::NAT*, pTO154 for *ELM1::NAT*, pTO169 for *Δace2* + *ACE2*, pTO174 for *Δtao3* + *TAO3*, and pTO175 for *Δelm1* + *ELM1* using primers oTO18-oTO19. To generate *Δtao3*, a linear repair cassette incorporating 50–70 bp homology to either end of the target gene flanking the NAT cassette was amplified from pTO137 using primers oTO353-oTO354. Linear sgRNA cassettes were amplified from pTO136 using fusion PCR as described previously to replace the gRNA sequence with gRNA targeting each gene for deletion^20^. Fusion fragments were amplified using primers oTO333-oTO225 and oTO224-oTO334 to target *ELM1*, oTO335-oTO225 and oTO224-oTO336 to target *ACE2*, oTO356-oTO224 and oTO355-oTO225 to target *TAO3*, and oTO224-oTO519 and oTO225-oTO518 to target *NAT*. Each pair of fragments with overlapping sequences were spliced on extension using oTO18-oTO19. PCR products were purified with a Zymo DNA Clean & Concentrator kit (Cat no. D4034, Zymo Research) according to the manufacturer’s instructions. *C. auris* cells were incubated overnight at 30 °C in YPD to exponential phase, not exceeding OD_600_ of 2.2. Cells were harvested by centrifugation and resuspended in TE buffer with 100 mM lithium acetate then incubated with constant shaking at 30 °C for 1 h. DTT was added to the cells at a final concentration of 25 mM and incubation was continued for 30 min at 30 °C with constant shaking. The cells were harvested by centrifugation; washed once with ice-cold, sterile, ultrapure water; washed once with ice-cold 1 M sorbitol; then resuspended in ice-cold 1 M sorbitol. 40 μL of competent cells was added to a pre-chilled 2-mm-gap electro-cuvette along with 1 μg each of the PCR-amplified linear transformation cassettes encoding Cas9, sgRNA, and the repair cassette. Alternatively, to compare targeted integration efficiency, an equal volume of Zymo elution buffer was added instead of Cas9 or sgRNA cassettes. Cells were electroporated using a Bio-Rad MicroPulser Electroporator set to the programmed *P. pastoris* (PIC) protocol (2.0 kV, 1 pulse), recovered in 1 M sorbitol, then resuspended in YPD and allowed 2 h of outgrowth at 30 °C with shaking. The cells were then spread-plated on YPD with 200 μg/mL nourseothricin and incubated at 30 °C or 1000 μg/mL G418 and incubated at 23 °C. Mutant strains were confirmed with PCR and Sanger sequencing and were confirmed to not exhibit stable integration of the *CAS9* cassette using *CAS9-*specific PCR primers oTO514-oTO515.

To estimate the efficiency of targeted RFP integration among transformant colonies, transformation plates were imaged using a Typhoon FLA 9500 Bioimager fitted with a 532 nm filter. Fluorescent images were visualized using Fiji Software^63^. An intensity threshold was set to identify transformant colonies exhibiting fluorescence. Five representative fluorescent colonies and five representative non-fluorescent colonies from transformations performed in AR0387 were spotted onto YPD agar and grown at 30 °C for 2 days. A sample of the colony growth was collected from each colony and suspended in 15 μL water. An aliquot of this suspension was used as a template in PCR reactions with primers overlapping the junction of the predicted *ENO1*-*RFP* insertion site or a genomic region upstream of the junction present in the wild-type locus. Colony PCR was performed using Phire Plant Direct PCR Master Mix (F160S; Thermo Fisher Scientific) according to the manufacturer’s instructions. The proportion of transformant colonies with targeted integration was determined by dividing the number of colonies exhibiting fluorescence by the total number of transformant colonies.

### Live cell microscopy

Cells were grown to mid-exponential phase at 30 °C in YPD and pelleted by centrifugation for 1 min at 4000 rpm (1500 × *g*) then resuspended in PBS. 5 μL cell suspension was combined with 1 μL of 0.1 g/L Calcofluor White stain and applied to a glass microscope slide. Alternatively, overnight cultures were prepared for each isolate and wild-type strain in yeast extract peptone dextrose (YPD) at 30 °C, with rotation, and then subcultured to mid-log phase before washing twice in 1× PBS and staining with FM4−64 (BioTracker 640 Red C2(FM4−64) Synaptic Dye, Millipore Sigma) at 10 µM for 10 min. Cells were visualized using an Olympus IX70 Epifluorescent Microscope fitted with a Hamamatsu C11440 camera and taken with Olympus CellSens v. 3.2 software.

### Stereomicroscopy

*C. auris* cells were grown on YPD agar at 30 °C for 2–7 days to form colonies. Colonies were visualized using a Leica KL300 LED stereomicroscope.

### RNA extraction

RNA extraction was performed as described previously^64^. Briefly, cells were grown to mid-exponential phase at 30 °C in YPD and harvested by centrifugation. Cells were washed in PBS, then centrifuged and all liquid removed. Dry cell pellets were frozen on dry ice then stored at −80 °C overnight. Cell pellets were thawed and resuspended in 100 μL FE Buffer (98% formamide, 0.01 M EDTA) at room temperature. 50 μL of 500 μm RNAse-free glass beads was added and the cell suspension was ground in three cycles of 30 s using a BioSpec Mini-Beadbeater-16 (Biospec Products Inc., Bartlesville, OK, USA). The cell lysate was centrifuged to remove cell debris and the crude RNA extract collected from the supernatant. The extract was DNAse-treated and purified using a Qiagen RNeasy mini kit (ref. 74104, Qiagen) as per the manufacturer’s instructions. RNA integrity was confirmed through agarose gel electrophoresis using the bleach gel method^65^.

### RT-qPCR

cDNA was synthesized from isolated RNA using the AffinityScript qPCR cDNA Synthesis Kit (ref. 600559, Agilent Technologies) according to the manufacturer’s instructions and used as a template for qPCR. qPCR was performed in three biological replicates, each with three technical replicates using a Bio-Rad CFXConnect Real Time System. Fold changes were calculated using the double-delta CT method with expression normalized to that of *ACT1* and compared to wild type. Amplification was measured for *ACT1* using primers oTO359-oTO360, for *CHS2* using primers oTO361-oTO362, and for *CTS1* using primers oTO363-oTO364.

### Co-expression genetic interaction analysis

The *C. albicans* ortholog of *B9J08_002252* was identified through orthology on the Candida Genome Database as C7_00260C_A. This was used as a query in CalCEN and the top 50 most co-expressed neighbors were identified. This set was then examined for putative function through GO term enrichment in the Candida Genome database. The network was visualized using Cytoscape.

### *Galleria mellonella* infections

Infections were performed as previously described^66^, with minor modifications. Briefly, *G. mellonella* larvae were purchased from *speedyworm.com* and maintained in sawdust at room temperature. Overnight *C. auris* cultures were prepared for each isolate and wild-type strain in yeast extract peptone dextrose (YPD) at 30 °C, with rotation. We were unable to standardize inoculum by direct cell count between aggregating and non-aggregating strains, so we standardized each inoculum to a consistent optical density. Cells were washed twice in PBS and diluted to an OD600 of 1 in 1× PBS, and 50 µL was inoculated into the larvae using an exel veterinary U-40 diabetic syringe (0.5CC × 29G × ½). Twenty larvae were infected per *C. auris* strain. After injection, larvae were maintained at 37 °C and monitored daily for survival. Virulence was analyzed using Kaplan−Meier survival curves in GraphPad Prism (version 9).

### Antifungal susceptibility assays

*C. auris* colonies were suspended in PBS to OD_600_ = 1.0. A sterile cotton applicator was saturated with the cell suspension and used to inoculate the entire surface of a YPD plate three times, rotating the plate approximately 60° each time. The surface of the agar was allowed to dry at room temperature. MIC test strips containing 0.016–256 mg/L Fluconazole (Liofilchem REF 921471), 0.002–32 mg/L Caspofungin (Liofilchem REF 921541), or 0.002–32 mg/L Amphotericin B (Liofilchem REF 921531) were placed onto the surface of the agar. Plates were incubated inverted at 37 °C for 24 h and MICs were determined at the intersection between the zone of inhibition and the MIC test strip gradient.

### Statistics and reproducibility

Statistical analyses were carried out using R statistical software (version 4.3) or GraphPad Prism (version 9). Data are presented as means ± standard error of means from biological replicates. Except where otherwise specified, each experiment was performed in at least three independent biological replicates yielding similar results. Statistical significance among different groups was calculated using one-way ANOVA, with Tukey’s or Dunnett’s post hoc tests for multiple comparisons, chi-square test, or Mantel−Cox log-rank test. *p ≤ 0.05; **p ≤ 0.01; ***p ≤ 0.001; n.s., *p* > 0.05.

### Reporting summary

Further information on research design is available in the Nature Research Reporting Summary linked to this article.

## Supplementary information

Supplementary Information

Reporting Summary

## Data Availability

Data from Illumina sequences used to identify transgene insertion sites are available in the NCBI SRA under BioProject accession number PRJNA722500. Gene sequences for mapping and designing constructs were retrieved from the B8441 genome assembly (NCBI GCA_002759435.2) through fungidb.org. Source data are provided with this paper.

## Source data

Source Data
