# Author Correction: Forward and reverse genetic dissection of morphogenesis identifies filament-competent *Candida auris* strains

**DOI:** 10.1038/s41467-022-28739-1

**Published:** 2022-02-23

**Authors:** Darian J. Santana, Teresa R. O’Meara

**Affiliations:** Department of Microbiology and Immunology, University of Michigan Medical School, Ann Arbor, MI USA

**Keywords:** Fungal biology, Fungal genetics, Cellular microbiology, Pathogens

Correction to: *Nature Communications* 10.1038/s41467-021-27545-5, published online 10 December 2021.

The original version of this Article contained an error in Fig. 4a, in which the CFW image of the ‘*Δace2* + *ACE2*’ strain was inadvertently duplicated and incorrectly used as CFW image for the ‘*Δtao3* + *TAO3*’ strain. This has been corrected in both the PDF and HTML versions of the Article.

