# Supplementary material for: Forward and reverse genetic dissection of morphogenesis identifies filament-competent Candida auris strains
Source: Nat Commun. 2021 Dec 10;12:7197. doi: 10.1038/s41467-021-27545-5 (PMC8664941; doi:10.1038/s41467-021-27545-5)
Supplement: Supplementary file 1 — Supplementary Information [file 41467_2021_27545_MOESM1_ESM.pdf]

Supplementary Figure 1 A *C. albicans* ortholog of *B9J08_002252* is coexpressed with genes involved in piecemeal autophagy of the nucleus. For the *C. albicans* gene *C7_00260C*, a putative ortholog of the *C. auris* gene *B9J08_002252*, coexpressed genes were identified and analyzed for GO term association using the CalCEN coexpression network. Each node represents an individual gene and each edge corresponds to the relative degree of coexpression. 43 of 50 coexpressed genes fall under the "Piecemeal autophagy of the nucleus" GO term (dark green) and 7 fall under "GO term unknown, no annotation available" (light blue).

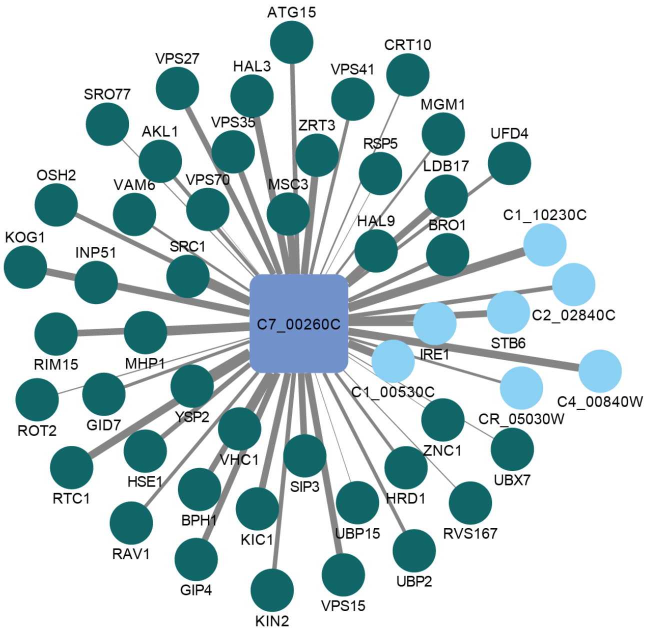
C7_00260C
ATG15
CRT10
HAL3
VPS41
VPS27
MGM1
SRO77
UFD4
ZRT3
AKL1
VPS35
RSP5
LDB17
OSH2
VAM6
VPS70
MSC3
C1_10230C
HAL9
KOG1
INP51
SRC1
BRO1
C2_02840C
STB6
IRE1
RIM15
MHP1
C1_00530C
C4_00840W
ROT2
GID7
YSP2
CR_05030W
ZNC1
VHC1
RTC1
HSE1
UBX7
SIP3
HRD1
BPH1
KIC1
UBP15
RVS167
RAV1
UBP2
GIP4
VPS15
KIN2

Supplementary Figure 2 A sixth insertional mutant identified with irregular morphology through AtMT. DIC microscopy of a sixth insertional mutant that was sequenced in pool with mutants from Figure 2. Scale bar = 20 µm. The exact genomic locations of transgene insertion sites in this mutant could not be determined.

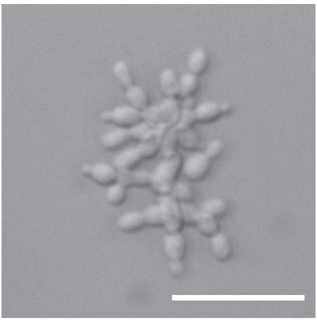

Supplementary Figure 3 *C. auris* isolates exhibit differential homology to the *ENO1* 3' homologous arm used in the targeted transformation efficiency reporter cassette. The sequence of the 3' homologous arm used in the cassette is provided. A pairwise alignment between this sequence and the genomic sequence corresponding to the homologous region in each of the four isolates tested for targeted transformation efficiency (AR387, AR381, AR383, AR386) indicates differences in homology to the reporter cassette. Homology at any given position is indicated by '.' while a nucleotide polymorphism at any given position is indicated by A, T, C, or G.

```
Eno1_3'    1 GTTTGCGCTTCAAACCACTTTGGTATATAATCTAGACGAAATATCATACTAAGAACGTTA
AR387      1 ............................................................
AR381      1 ............................................................
AR383      1 ............................................................
AR386      1 ............................................................
Eno1_3'   61 CAATGCAGTAGATATGTACAAGACTAGCTTCTCGAAAGCTCATCTAAAGCCTCAACTATT
AR387     61 ............................................................
AR381     61 ............................................................
AR383     61 ............................................................
AR386     61 ............................................................
Eno1_3'  121 CTCTTTACCACGACTCTCACATCCTCAAGAGTATTGTACAATGGTGTGGGTGCAATACGA
AR387    121 ............................................................
AR381    121 ............................................................
AR383    121 ............................................................
AR386    121 ............................................................
Eno1_3'  181 ATAACATCGGGTCTCCTCTCGTCGCAAATGATTGCATGCTTGTGTAAGTACTCGTTTACC
AR387    181 ............................................................
AR381    181 ............................................................
AR383    181 ............................................................
AR386    181 ............................................................
Eno1_3'  241 TGTTCCATAACATTCTTGCTTCTGTCGTCATCATGCGGTTGGAATAACACTGAAAGTTGA
AR387    241 ............................................................
AR381    241 ............................................................
AR383    241 ............................................................
AR386    241 ............................................................
Eno1_3'  301 CATCCTCTCTCACTTTCTTCACATGGAGTCAAGATCTTGAATCCCAACAGTGCATTGTCC
AR387    301 ............................................................
AR381    301 ............................................................
AR383    301 ............................................................
AR386    301 ............................................................
Eno1_3'  361 AGCGGATCTTCGATGTAGTATTTGCTGCCCTTCAAAAGCCTTAGCAAGCAGCCAGTCAAA
AR387    361 ............................................................
AR381    361 .......................................G....................
AR383    361 .......................................G....................
AR386    361 ............................................................
Eno1_3'  421 GAAACACTAGTTTTTCTAAGATTATCCAACCCTCCCGCTAGTTCAAAAACATCAAGAGAA
AR387    421 ............................................................
AR381    421 .......................G...................................G
AR383    421 .......................G...................................G
AR386    421 .......................G....................................
Eno1_3'  481 CTCTTCAATGCAACACAGTCAATCACCAGCGGGTTAGATTGACGATAGGAGAGGGCAGAG
AR387    481 ............................................................
AR381    481 ................................A...........................
AR383    481 ................................A...........................
AR386    481 ............................................................
Eno1_3'  541 TTGATTGGGTCAAAGAC
AR387    541 .................
AR381    541 .................
AR383    541 .................
AR386    541 .................
```

Supplementary Figure 4 Detection of integration of the *CAS9* expression cassette in *C. auris* following transformation. Transformation was performed in *C. auris* isolates from each of the four major clades using the *CAS9* and sgRNA expression cassettes and the *ENO1-RFP* reporter cassette. Eight representative transformants were recovered from among those that exhibited robust fluorescence, indicating targeted integration, for each isolate. Detection of stable integration of *CAS9* was measured using PCR primers specific to the *CAS9* expression cassette. c, pTO135 was used as template in the PCR reaction.

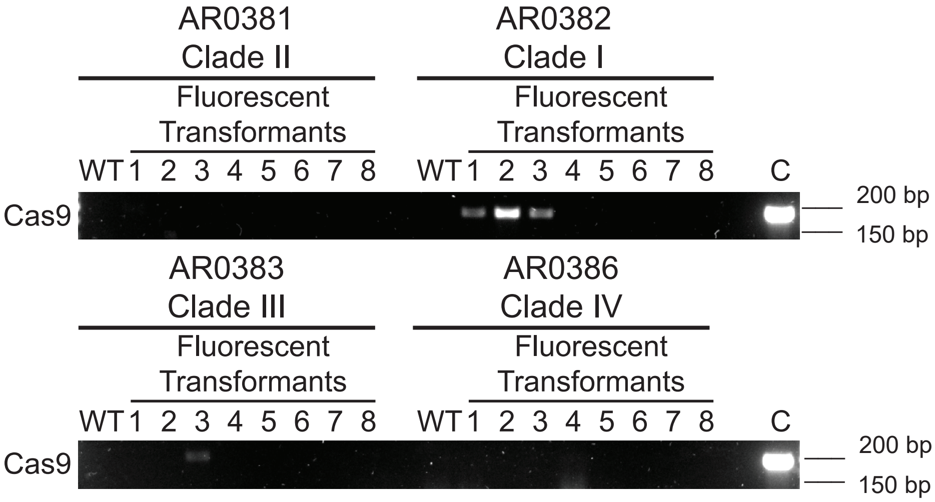

AR0381
Clade II
Fluorescent
Transformants
AR0382
Clade I
Fluorescent
Transformants
WT 1 2 3 4 5 6 7 8
WT 1 2 3 4 5 6 7 8
C
Cas9
200 bp
150 bp
AR0383
Clade III
Fluorescent
Transformants
AR0386
Clade IV
Fluorescent
Transformants
WT 1 2 3 4 5 6 7 8
WT 1 2 3 4 5 6 7 8
C
Cas9
200 bp
150 bp

Supplementary Figure 5 *C. auris* morphogenic mutants virulence profiles in *G. mellonella* infection model. Wild type AR0382 (Clade I), *Δtao3* and *Δtao3* + *TAO3* strains (a) or *Δace2* and *Δace2* + *ACE2* strains (b) in the AR0382 background were standardized to an optical density of OD_{600} = 1.0 in PBS before inoculating 20 *Galleria mellonella* larvae per *C. auris* strain with 50 µL of prepared inoculum. Larvae were maintained at 37 °C and monitored daily for survival for 5 days. Statistical differences were determined between survival curves using a Mantel-Cox log-rank test: (a) WT-*Δtao3*, ns: p = 0.0563; WT-*Δtao3* + *TAO3,* ns: p = 0.7899; *Δtao3*-*Δtao3* + *TAO3*, *: p = 0.0275 (b) WT-*Δace2*, *: p = 0.0427; WT-*Δace2* + *ACE2,* ns: p = 0.8962; *Δace2*-*Δace2* + *ACE2,* *: p = 0.0158.

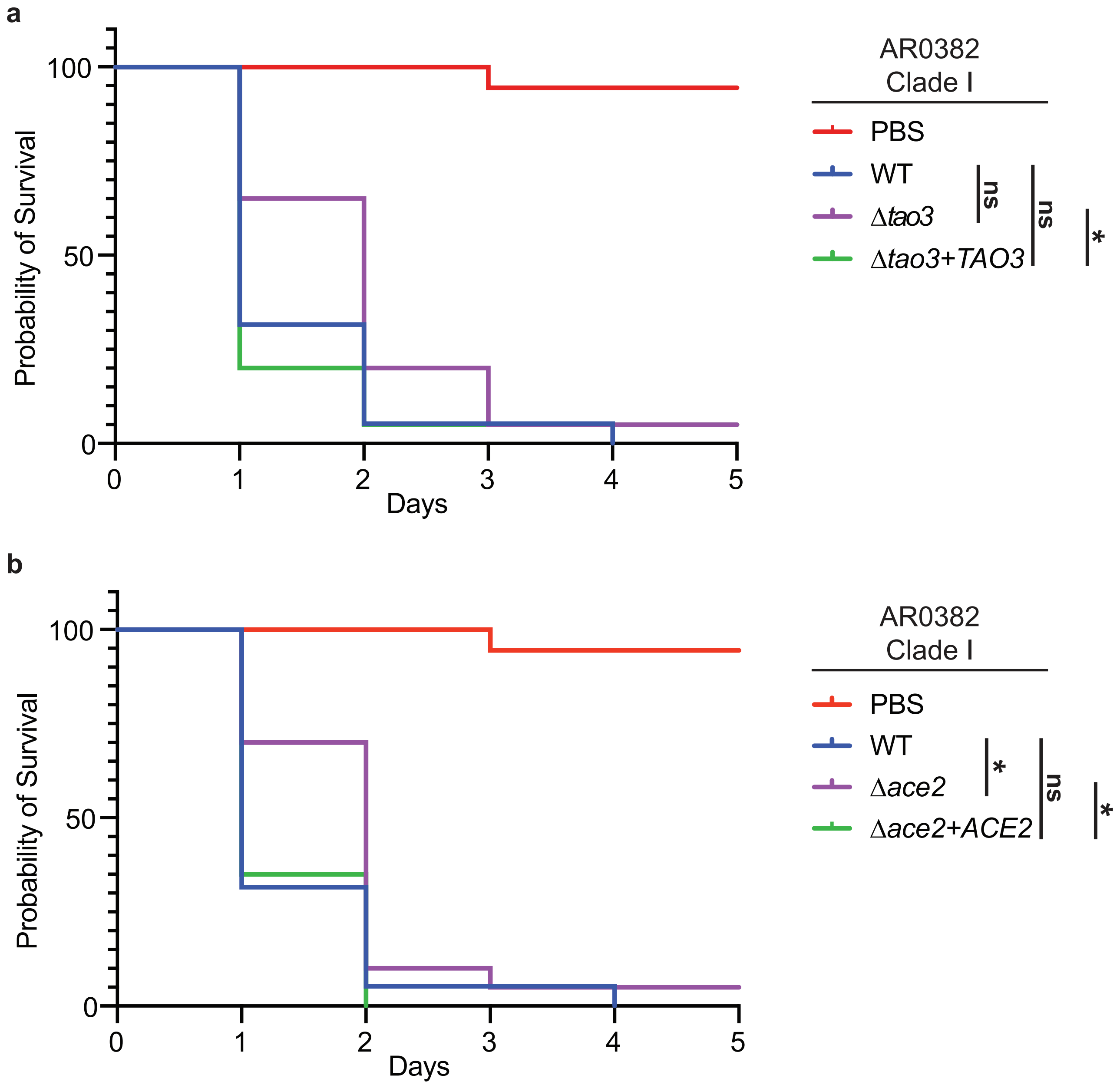
a
Probability of Survival
100
50
0
0
1
2
3
4
5
Days
AR0382
Clade I
PBS
WT
Δtao3
Δtao3+TAO3
ns
ns
*
b
Probability of Survival
100
50
0
0
1
2
3
4
5
Days
AR0382
Clade I
PBS
WT
Δace2
Δace2+ACE2
*
ns
*

Supplementary Table 1. MIC Values for AR0382, morphogenic mutants, and complemented strains

	MIC (mg/L)		
	Fluconazole	Caspofungin	Amphotericin B
AR0382	12	0.094	0.38
Δace2	8	0.125	0.38
Δace2 + *ACE2*	12	0.094	0.38
Δtao3	>256	0.064	1
Δtao3 + *TAO3*	12	0.125	0.38
Δelm1	32	0.023	0.38
Δelm1 + *ELM1*	8	0.125	0.38

Supplementary Table 2. Strains used in this study.

Strain Name	Alias	Genotype	Source
pTO123	*Agrobacterium tumefaciens* EHA105	Harbors pEHA105 (pTiBo542ΔT-DNA)	[1]
pTO131	*Agrobacterium tumefaciens* EHA105	Harbors pEHA105 + pTO128 (pPZP-NATca)	This Study
AR0381	*Candida auris* B11220		[2,3]
AR0382	*Candida auris* CDC-0382		[2,3]
AR0383	*Candida auris* B11221		[2,3]
AR0386	*Candida auris* B11245		[2,3]
AR0387	*Candida auris* B8441		[2,3]
CauTO58	*Candida auris* AR0382	Tn*ACE2*	This Study
CauTO112	*Candida auris* AR0382	TnB9J08_002252	This Study
CauTO113	*Candida auris* AR0382	Tn*CHS2*	This Study
CauTO114	*Candida auris* AR0382	Tn*ELM1*	This Study
CauTO115	*Candida auris* AR0382	Tn*TAO3*	This Study
CauTO180	*Candida auris* AR0382	*Δace2*	This Study
CauTO182	*Candida auris* AR0382	*Δelm1*	This Study
CauTO183	*Candida auris* AR0382	*Δtao3*	This Study
CauTO203	*Candida auris* AR0382	*Δace2 + ACE2*	This Study
CauTO216	*Candida auris* AR0382	*Δelm1 + ELM1*	This Study
CauTO217	*Candida auris* AR0382	*Δtao3 + TAO3*	This Study
CauTO184	*Candida auris* AR381	Δ*CauACE2*	This Study
CauTO185	*Candida auris* AR381	Δ*CauELM1*	This Study

Supplementary Table 3. Plasmids used in this study.

Name	Description	Source
pPZP-NEO1	*neoR* in T-DNA region, *kanR*	[4]
pTO128 (pPZP-NATca)	*CaNAT1R* in T-DNA region, *kanR*	This study
pLC49	*FLP-NAT, ampR, natR*	[5]
pLC963	*CaCas9,sgRNA, ampR*	[6]
pLC1047	*RFP-FLP-NAT, natR ampR*	[7]
pUC19	*ampR*	[8]
pTO135	*pENO1-CaCas9-tCyc1, ampR*	This study
pTO136	*pADH1-tRNA-Ala-gRNA-tracrRNA-HDV-tAgTEF2, ampR*	This study
pTO137	*RFP-tADH1-pAgTEF2-NAT1-tAGTEF2,* with targeting arms to tag Eno1 cterminus, *natR ampR*	This study
pTO154	*CauELM1::NAT, ampR natR*	This study
pTO155	*CauACE2::NAT, ampR natR*	This study
pTO149	*RFP-tADH1-pAgTEF2-NEO-tAGTEF2,* with targeting arms to tag Eno1 cterminus, *natR ampR*	This study
pTO169	*ace2 + ACE2, NEO*	This study
pTO174	*tao3 + TAO3, NEO*	This study
pTO175	*elm1 + ELM1, NEO*	This study

Supplementary Table 4. Oligonucleotides used in this study.

Name	Sequence	Use
oTO112	ttcgagctcggtaccCCTCTTTGTAGTTCAACTTATG	Construction of pTO135
oTO113	atactttttatccatGATGAAAATTAAGTTTGGATAGG	
oTO114	aacttaattttcatcATGGATAAAAAGTATAGTATTGGTTTAG	
oTO115	gactctagaggatccGTCCCAAAACCTTCTCAAG	
oTO116	agaaggttttgggacGGATCCTCTAGAGTCGAC	
oTO117	tgaactacaaagaggGGTACCGAGCTCGAATTC	
oTO118	ttcgagctcggtaccCGAGATAGATCGAAATACG	Construction of pTO136
oTO119	cgctaccaactacgccacacgcccgagtagGATTTCGTGAAGATTGATTG	
oTO120	gtcgattcgatactaacgccgccatccagtGGATCCTCTAGAGTCGAC	
oTO121	tttcgatctatctcgGGTACCGAGCTCGAATTC	
oTO122	ttcgagctcggtaccGGTGAGCAATTGGCTGAC	Construction of pTO137
oTO123	tgaaacaccaccaccCAAGTTTTGAGCAGCCTTG	
oTO124	gctgctcaaaacttgGGTGGTGGTGTTTCAAAAG	
oTO125	taatcatgctgctacTTATTTATATAATTCATCCATACCACC	
oTO126	gaattatataaataaGTAGCAGCATGATTATGAAC	
oTO127	cgaggcaagcttgatCCTAAACTGCAAACCCATC	
oTO128	ggtttgcagtttaggATCAAGCTTGCCTCGTCC	
oTO129	gtttgaagcgcaaacACTGGATGGCGGCGTTAG	
oTO130	acgccgccatccagtGTTTGCGCTTCAAACCAC	
oTO131	gactctagaggatccGTCTTTGACCCAATCAAC	
oTO132	gattgggtcaaagacGGATCCTCTAGAGTCGAC	
oTO133	agccaattgctcaccGGTACCGAGCTCGAATTC	
oTO317	TGAATTCGAGCTCGGTACCCCGTTTGGATAACAAAAACTCG	Construction of pTO154
oTO318	tactaacgccgccatccagtTGCCCTTTATGTTGCTTTCA	
oTO319	TGAAAGCAACATAAAGGGCAactggatggcggcgttagta	
oTO320	CAGATTCTCACTAAGCCTGCatcaagcttgcctcgtcccc	
oTO321	ggggacgaggcaagcttgatGCAGGCTTAGTGAGAATCTG	
oTO337	AGGTCGACTCTAGAGGATCCCTTTTCTTGAACGGAGGTAAT	
oTO323	TTACCTCCGTTCAAGAAAAGGGATCCTCTAGAGTCGACCT	
oTO324	GAGTTTTTGTTATCCAAACGGGGTACCGAGCTCGAATTCA	
oTO325	GTGAATTCGAGCTCGGTACCCCTTCTCTAGTTCAGGGTCCC	Construction of pTO155
oTO326	tactaacgccgccatccagtAGCGGGCGCTGGTGAAATTT	
oTO327	AAATTTCACCAGCGCCCGCTactggatggcggcgttagta	
oTO328	AAGGAAAAAGAAATGCAACGatcaagcttgcctcgtcccc	
oTO329	ggggacgaggcaagcttgatCGTTGCATTTCTTTTTCCTTT	
oTO330	AGGTCGACTCTAGAGGATCCCCAACTAATCCCAAAGGCCG	
oTO331	CGGCCTTTGGGATTAGTTGGGGATCCTCTAGAGTCGACCT	
oTO332	GGACCCTGAACTAGAGAAGGGGTACCGAGCTCGAATTCAC	
oTO272	ttcttgacgagttcttctgaTAACTCGAGTCAGTACTG	Construction of pTO149
oTO273	tcttgttcaatcatagacatAATTGTAGGATCCGGTTG	
oTO564	ctgttttacaggcGGATCCTCTAGAGTCGAC	Construction of pTO169
oTO565	tcgaaagagactcGGTACCGAGCTCGAATTC	
oTO566	gagctcggtaccGAGTCTCTTTCGATTTTGGGTGTTGAG	

oTO567	tcatgctgctacTCAACGTCTCTGGGCGGC	
oTO568	ccagagacgttgaGTAGCAGCATGATTATGAACAGG	
oTO569	aagaaatgcaacgACTGGATGGCGGCGTTAG	
oTO570	ccgccatccagtCGTTGCATTTCTTTTTCC	
oTO571	tctagaggatccGCCTGTAAAACAGGAGTC	
oTO582	ttggtgtcgatccggatcctctagagtcgac	Construction of pTO174
oTO583	taatcttgcatcgggtaccgagctcgaattc	
oTO584	gagctcggtacccgatgcaagattatctgc	
oTO585	tcatgctgctactcacttccgattctttttc	
oTO586	gaatcggaagtgagtagcagcatgattatgaacagg	
oTO587	ttgcaaattttccactggatggcggcgttag	
oTO588	ccgccatccagtggaaaatttgcaaccatttttctg	
oTO589	tctagaggatccggatcgacaccaaatggc	
oTO590	ggatcctctagagtcgac	Construction of pTO175
oTO591	ggtaccgagctcgaattc	
oTO592	ggccagtgaattcgagctcggtaccccttcaacacaaccgatag	
oTO593	tcatgctgctacttagtttttttcgagtctgtc	
oTO594	cgaaaaaaactaagtagcagcatgattatgaacagg	
oTO595	tcactaagcctgcactggatggcggcgttag	
oTO596	ccgccatccagtgcaggcttagtgagaatc	
oTO597	cctgcaggtcgactctagaggatccggaatgtctgcgtggattg	
oTO18	CAGGAAACAGCTATGAC	Amplification of transformation cassettes
oTO19	GTAAAACGACGGCCAG	
oTO353	GGAATCACCATTTTTAATCTCCCCTTCAGTAATTCACCTCCTTCT TCCTCTTTTCactggatggcggcgttag	*tao3::NAT* repair cassette
oTO354	GGTAGATATGAGGCCCTGCATAGTCGCGGAGTATAGACA ATTCATGCAGAAAAATGGTTGCAAATTTTCCatcaagcttgcctcgtcc	
oTO224	GCTATTACGCCAGCTGG	sgRNA Fusion PCR
oTO225	CGCAATTAATGTGAGTTAGC	
oTO333	GACTAGACATAGAGCTTGATgttttagagctagaaatagcaag	Fusion PCR – *ELM1* gRNA
oTO334	ATCAAGCTCTATGTCTAGTCTGGACGAGTCCGGATTC	
oTO335	CTCAACGAAACCTCGTACACgttttagagctagaaatagcaag	Fusion PCR – *ACE2* gRNA
oTO336	GTGTACGAGGTTTCGTTGAGTGGACGAGTCCGGATTC	
oTO355	TTGGTACAGGGAAACACAATgttttagagctagaaatagcaag	Fusion PCR – *TAO3* gRNA
oTO356	ATTGTGTTTCCCTGTACCAAtggacgagtccggattc	
oTO518	catctgggcagatgatgtcggttttagagctagaaatagcaag	Fusion PCR – *NAT gRNA*
oTO519	cgacatcatctgcccagatgtggacgagtccggattc	
oTO514	atagtttgggacaaagggcgt	Detection of *CAS9* Integration
oTO515	ggttggtgagtcgaaaccac	
oTO359	CGTGCTGTGTTCCCATCCAT	*ACT1* qPCR
oTO360	AGCCTCATCACCGACATACG	
oTO361	GAAACGGACGTGCCTGAAAG	*CHS2* qPCR
oTO362	TGCCGCAATGAGTAAAGTGC	
oTO363	GACTTGTCAGTCCAAGGGCA	*CTS1* qPCR
oTO364	AAGGTCTCTCGGAGTCGGAA	

oTO310	CCGAAACTATCTACATGACCC	AtMT Transgene Insertion Site Mapping
oTO311	CAGTCTCCATTTGCCTCTC	
oTO312	CCGATACACCAACATTGC	
oTO313	GCCATCGTTGCTAATCTTC	
oTO314	CTCCTTTCAGACATGCAAAG	
oTO315	GTGGTGGTCCTAACAGAG	
oTO316	CAACCATTTGTGTCTGTGC	
oTO338	GTTGGACCTCTGATCAGTATC	
oTO339	CTTTGAGGTAGGGTAGGAC	
oTO340	GTAGGAGTATTGGACCTCG	
oTO341	GGCATTGTAACAGTCTGAG	
oTO342	GATTCTCACTAAGCCTGC	
oTO343	GGTGCGCATAGATAAGG	
oTO344	GTGAAGACTACGCAAAGCATG	
oTO6	gactgtcaaggagggtattc	
oTO90	gctttatacgatggtactgc	

Supplementary References

1. Hood, E. E., Gelvin, S. B., Melchers, L. S. & Hoekema, A. New Agrobacterium helper plasmids for gene transfer to plants. Transgenic Res. 2, 208–218 (1993).
2. Lockhart, S. R. et al. Simultaneous Emergence of Multidrug-Resistant Candida auris on 3 Continents Confirmed by Whole-Genome Sequencing and Epidemiological Analyses. Clin. Infect. Dis. 64, 134–140 (2017).
3. Lutgring, J. D. et al. FDA-CDC Antimicrobial Resistance Isolate Bank: a Publicly Available Resource To Support Research, Development, and Regulatory Requirements. J. Clin. Microbiol. 56, (2018).
4. Walton, F. J., Idnurm, A. & Heitman, J. Novel gene functions required for melanization of the human pathogen Cryptococcus neoformans. Mol. Microbiol. 57, 1381–1396 (2005).
5. Cowen, L. E. et al. Harnessing Hsp90 function as a powerful, broadly effective therapeutic strategy for fungal infectious disease. Proc. Natl. Acad. Sci. U. S. A. 106, 2818–2823 (2009).
6. Veri, A. O. et al. Tuning Hsf1 levels drives distinct fungal morphogenetic programs with depletion impairing Hsp90 function and overexpression expanding the target space. PLoS Genet. 14, e1007270 (2018).
7. O'Meara, T. R. et al. Global proteomic analyses define an environmentally contingent Hsp90 interactome and reveal chaperone-dependent regulation of stress granule proteins and the R2TP complex in a fungal pathogen. PLoS Biol. 17, e3000358 (2019).
8. Norrander, J., Kempe, T. & Messing, J. Construction of improved M13 vectors using oligodeoxynucleotide-directed mutagenesis. Gene vol. 26 101–106 (1983).
